# Understanding how different surfaces and environmental biofilms found in food processing plants affect the spread of COVID-19

**DOI:** 10.1371/journal.pone.0286659

**Published:** 2023-06-07

**Authors:** Austin Featherstone, Amanda Claire Brown, Sapna Chitlapilly Dass

**Affiliations:** Department of Animal Science, Texas A&M University, College Station, Texas, United States of America; Universidade Lisboa, Instituto superior Técnico, PORTUGAL

## Abstract

Meat processing plants have been at the center of the SARS-CoV-2 pandemic, with a recent report citing 90% of US facilities having multiple outbreaks during 2020 and 2021. We explored the potential for biofilms to act as a reservoir in protecting, harboring, and dispersing SARS-CoV-2 throughout the meat processing facility environment. To do this, we used Murine Hepatitis Virus (MHV), as a surrogate for SARS-CoV-2, and meat processing facility drain samples to develop mixed-species biofilms on materials found in meat processing facilities (stainless steel (SS), PVC, and ceramic tiles). After exposure to the biofilm organisms for five days post-inoculation at 7°C we conducted quantitative PCR (qPCR) and plaque assays to determine whether MHV could remain both detectable and viable. Our data provides evidence that coronaviruses can remain viable on all the surfaces tested and are also able to integrate within an environmental biofilm. Although a portion of MHV was able to remain infectious after incubation with the environmental biofilm, a large reduction in plaque numbers was identified when compared with the viral inoculum incubated without biofilm on all test surfaces, which ranged from 6.45–9.27-fold higher. Interestingly, we observed a 2-fold increase in the virus-environmental biofilm biovolume when compared to biofilm without virus, indicating that the biofilm bacteria both detected and reacted to the virus. These results indicate a complex virus-environmental biofilm interaction. Although we observed better survival of MHV on a variety of surfaces commonly found in meat processing plants alone than with the biofilm, there is the potential for biofilms to protect virions from disinfecting agents, which has implications for the potential of SARS-CoV-2 prevalence within the meat processing plant environment. Also given the highly infectious nature of SARS-CoV-2, particularly for some of the variant strains such as omicron, having even a residual level of virus present represents a serious health hazard. The increase in biofilm biovolume in response to virus is also a concern for food safety due to the potential of the same being seen with organisms associated with food poisoning and food spoilage.

## Introduction

Coronaviruses are a diverse group of positive-sense, enveloped, single-stranded RNA viruses [1]. Beta-coronaviruses are part of a diverse group of positive-sense, enveloped, single-stranded RNA viruses that exclusively infect mammals [1, 2]. To date, there are nine known human coronaviruses, of which, three are documented to be highly pathogenic and lethal: Middle-East Respiratory Syndrome Coronavirus (MERS-CoV), Severe Acute Respiratory Syndrome Coronavirus (SARS-CoV), and SARS-CoV-2 [3–5]. Prior to SARS-CoV-2, coronaviruses such as MERS-CoV and SARS-CoV could only be transmitted to humans through an animal reservoir [6–10]. Thus, SARS-CoV-2, the etiologic agent of Coronavirus Disease 2019 (COVID-19), spread across the world in a few months [11, 12]. Due to how quickly the virus spread at the beginning of 2020, and the severe number of COVID-19 cases seen around the world, many businesses had to temporarily close to help prevent the spread of COVID-19 [13–15].

Meat processing plants have been at the center of the SARS-CoV-2 pandemic, with a recent report citing 90% of US facilities experiencing multiple outbreaks during 2020 and 2021, most likely due to the environmental conditions inside of the meat processing plant being an excellent reservoir for SARS-CoV-2 and other harmful pathogens, such as biofilms, to be able to survive [16]. Biofilms are multicellular assemblages of prokaryotic and eukaryotic cells that are enclosed in a polysaccharide material [17, 18]. Bacterial and fungal biofilms have so far been a focus of research in food processing facilities, with biofilms considered a major threat to food safety, due to the carriage of foodborne pathogens [19, 20]. Research on the presence of virus particles in the mixed-species biofilm community is sparse, however, there are reports of biofilms protecting viruses from disinfectants, particularly chlorine [21], and the emergence of SARS-CoV-2 following colonization of a progenitor coronavirus from biofilms associated with bat habitats has been considered [22].

There are several factors that exist which allow us to consider biofilms as an ideal site to harbor SARS-CoV-2 in meat processing facilities. One reason is that meat processing facilities themselves are maintained at 4-7°C, and SARS-CoV-2 virions are both stable at colder temperatures and able to persist for several days on stainless steel (SS), copper, plastic, PVC and cardboard [23], all of which are materials commonly found in meat processing plants. Therefore, these facilities have a high potential for the harboring and transmitting SARS-CoV-2 [24]. While bacteria individually do not support virus infection, they can promote viral fitness as some viruses can use components of the bacterial envelope to enhance their stability [25–27]. As previously mentioned biofilms can also provide physical protection from disinfecting agents [21]. Moreover, bacterial communities and biofilms can impact the infection of mammals by viruses [25, 26, 28]. Furthermore, from a biophysics perspective, virus stability could also be enhanced by the thin liquid film produced by bacterial biofilms, preventing desiccation [23, 29].

To date, there is a critical gap of knowledge in understanding the stability and infectious state of SARS-CoV-2 in multi-species biofilms, particularly in meat processing plants. In this study, MHV was employed as a surrogate to elucidate the survival of coronaviruses within biofilms. MHV survival was analyzed by RT-qPCR and plaque assays following inoculation of MHV into meat processing plant environmental biofilms developed on SS, PVC, and ceramic tiles at 7°C.

## Results

### Mixed species biofilm cell numbers on all test surfaces are increased in the presence of MHV

**Fig 1:**
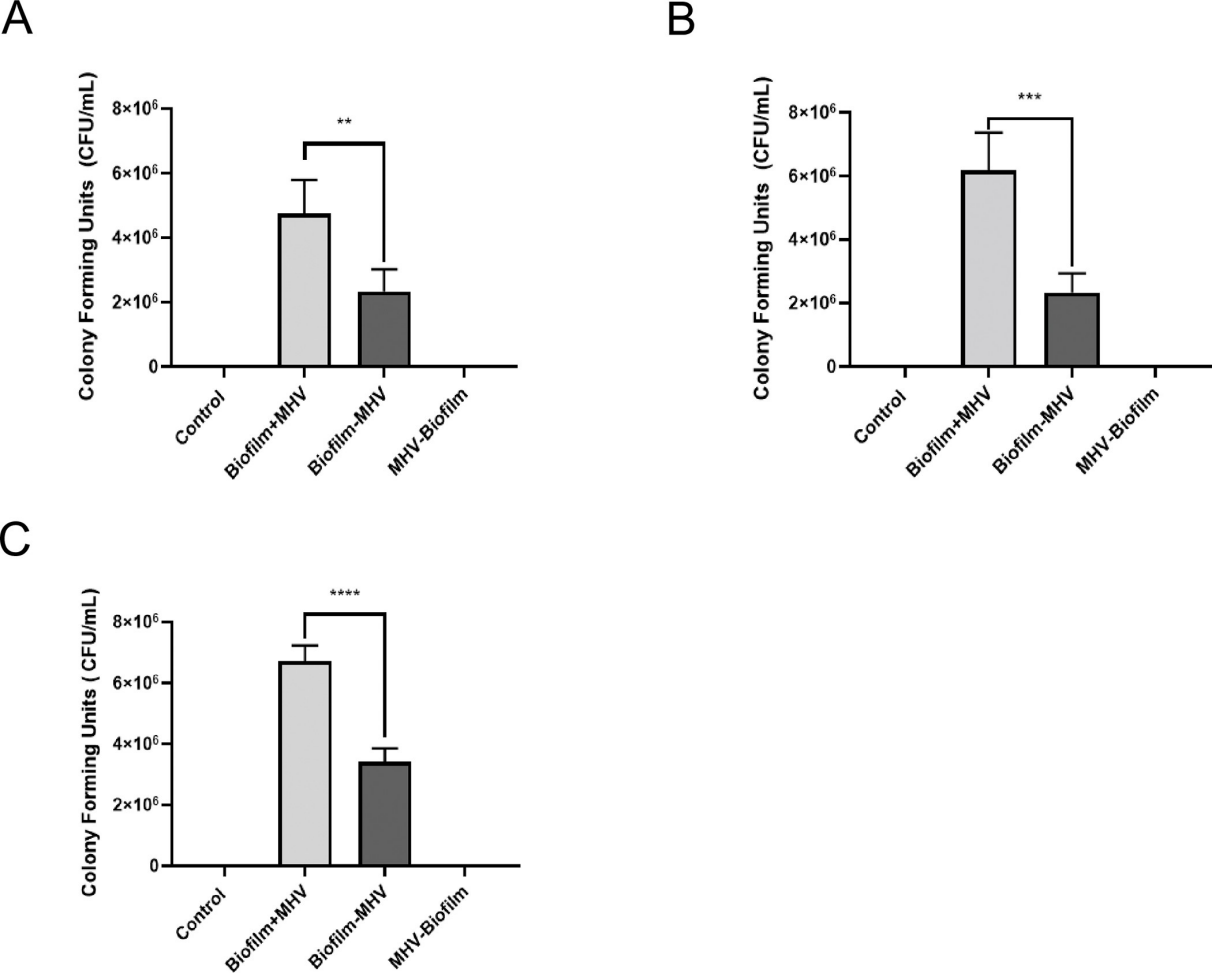
CFU counts from biofilm + MHV and biofilm samples on stainless steel, PVC, and tile chips. (A-C) CFU counts for biofilm + MHV and biofilm samples on (A) stainless steel, (B) PVC, and (C) ceramic tile chips. Each sample was plated in duplicate. Results in this figure are the mean values and standard deviations (error bars) from three independent experiments. Statistical significance was analyzed by unpaired t-test. **: *p* < 0.01; ***: *p* < 0.001; ****: *p* < 0.0001.

To determine if MHV influences the growth or decay of an environmental biofilm when inoculated together on different solid surfaces found in meat processing plants, we grew biofilms, consisting of environmental microorganisms obtained from a meat processing plant drain, on sterile SS (Fig 1A), PVC (Fig 1B), and ceramic tile chips (Fig 1C) and let the samples incubate for five days at 7°C (Table 1). Bacteria recovered from the Biofilm-MHV on SS samples ranged from 1.6 to 4.0x10^6^ CFU/mL. In comparison, the Biofilm+MHV on SS numbers ranged from 3.5 to 6.0x10^6^ CFU/mL (Fig 1A, Table 1), therein the presence of the virus generating a 1.74-fold increase in the biovolume of the biofilm. The CFU numbers for Biofilm-MHV exposed to PVC ranged from 1.8 to 4.0x10^6^ CFU/mL, whereas the number recovered from the Biofilm+MHV on PVC ranged from 5.0 to 7.8x10^6^ CFU/mL (Fig 1B, Table 1), representing a 2.10-fold increase in biovolume when MHV was present. Likewise, the overall mean biofilm microbial cells recovered from the Biofilm-MHV on ceramic tile ranged from 1.8 to 3.8x10^6^ CFUs/mL, while Biofilm+MHV on ceramic tile ranged from 4.8 to 7.0x10^6^ CFUs/mL (Fig 1C, Table 1), representing a 2.11-fold increase in biovolume, and very similar to what was seen with PVC. Therefore, our results indicate that MHV positively influences the growth of environmental microorganisms on SS, PVC, and on ceramic tiles.

**Table 1:** Data from the biofilm +/- MHV CFU/mL count from different experimental conditions. Table 1 indicates CFU/mL numbers and the percentage and fold change compared to the initial biofilm inoculum.

	CFU/mL for Biofilm + MHV on SS	CFU/mL for Biofilm + MHV on PVC	CFU/mL for Biofilm + MHV on Tile
Control	0; 0	0; 0	0; 0
Biofilm+MHV	5.25 x 10^6 (+191.7%, 2.92-fold); 4.25 x 10^6 (+49.1%, 1.49-fold).	5.35 x 10^6 (+189.2%, 2.89-fold); 7.00 x 10^6 (+150.0%, 2.50-fold).	6.50 x 10^6 (+83.1%, 1.83-fold); 6.90 x 10^6 (+112.3%, 2.12-fold).
Biofilm-MHV	1.80 x 10^6; 2.85 x 10^6	1.85 x 10^6; 2.80 x 10^6	3.55 x 10^6; 3.25 x 10^6
MHV-Biofilm	0; 0	0; 0	0; 0

### RNA recalcitrance on surfaces was less in the presence of biofilm

**Fig 2:**
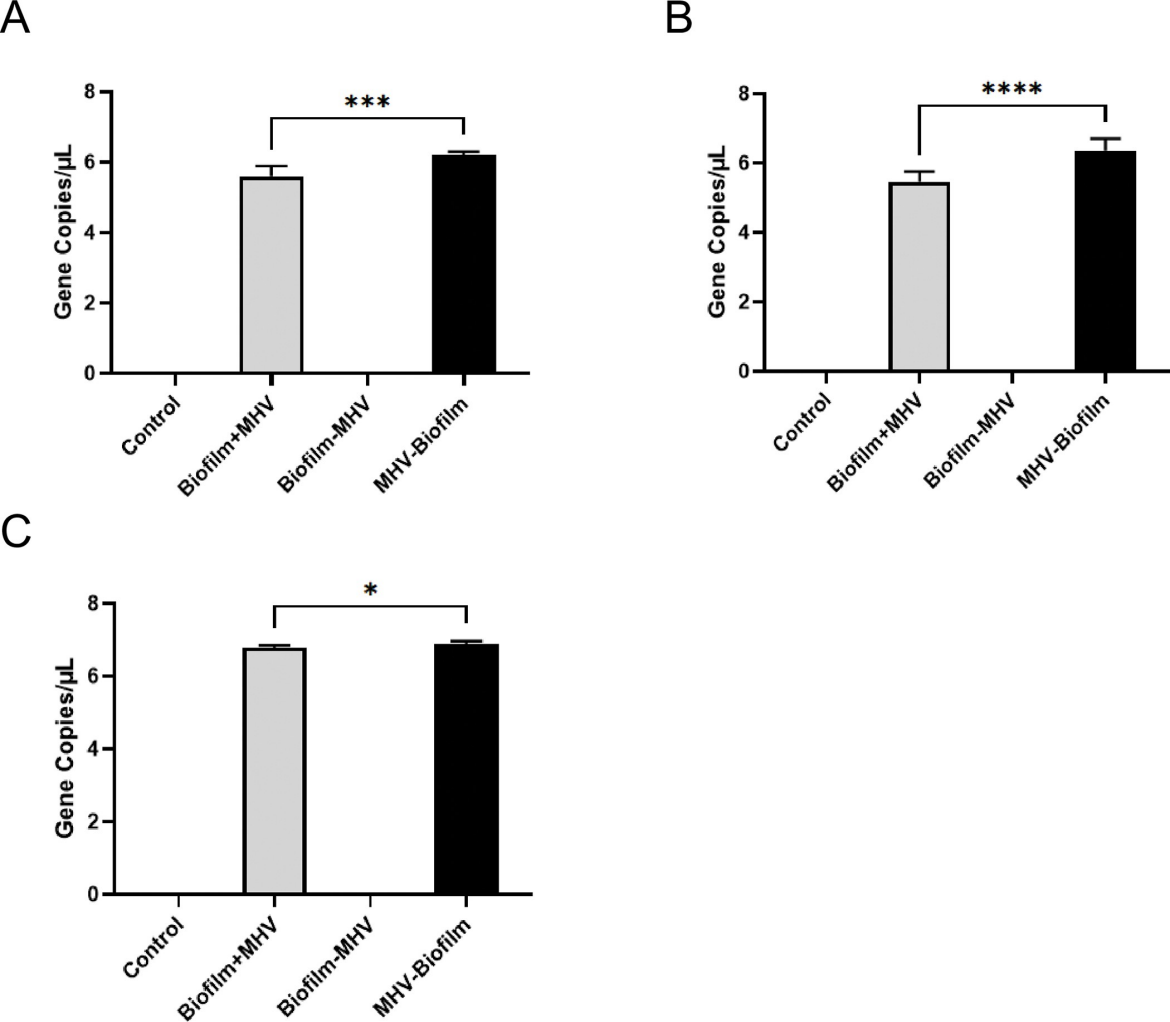
qPCR analysis of MHV mixed with biofilm and pre-incubated for 5 days on stainless steel, PVC, and ceramic tile chips. (A-C) qPCR analysis of MHV mixed with environmental biofilm on stainless steel, PVC and on tile chips. 1.0 x 10^3^ PFU of MHV were added to a SS, PVC, or ceramic tile chip along with a floor drain biofilm sample collected from the cooler of a single meat processing plant drain. The qPCR samples were analyzed in quadruplicate. Gene copy numbers were calculated from a standard curve of known quantities of MHV RNA in a 25 μL qPCR reaction. Results in this figure are the mean values and standard deviations (error bars) from two independent experiments. Statistical significance was analyzed by unpaired t-test. ns: not significant; *: *p* < 0.05; ***: *p* < 0.001, ****: *p* < 0.0001.

To determine whether there was a significant loss of intact MHV when incubated with an environmental biofilm on SS, PVC, and ceramic tile, we performed RT- qPCR analyses for the membrane gene (M) of MHV in our Biofilm + MHV, Biofilm-MHV, and MHV-Biofilm samples, as well as a negative control. Our RT-qPCR data revealed there was a statistically significant difference (unpaired T-test) in the persistence of viral RNA when a mixed species biofilm was present, compared with when the viral RNA was incubated on surfaces alone (Table 2). Specifically, the average gene copy numbers for the M-gene for Biofilm+MHV on SS was 5.60 gene copies/μL, compared with 6.21 gene copies/μL in the absence of biofilm (MHV-Biofilm, Fig 2A, Table 2). On PVC the average M-gene copy number for the Biofilm+MHV sample was 5.45 gene copies/μL, and 6.36 gene copies/μL for MHV-Biofilm (Fig 2B, Table 2), thus very similar to the trend we saw for SS. However, ceramic tile gave an average M-gene copy number for the Biofilm+MHV sample of 6.79 gene copies/μL, and MHV-Biofilm was 6.88 gene copies/μL (Fig 2C, Table 2). If we take each M-gene copy to equate to one virus/one plaque forming unit (PFU), our data indicate that 31.2–45.5% MHV was lost across all conditions when compared to the inoculum (100 μL at 1 x 10^4^ PFU, thus 10 PFU/μL). However, there was an overall loss we observed that viral RNA was more significantly degraded when exposed to the environmental biofilms on SS and PVC, interestingly only a marginal reduction was observed on ceramic tile in the presence of biofilm, but this was still statically significant *(p* = 0.05).

**Table 2:** Data from the biofilm +/- MHV RT-qPCR analyses on the recovered MHV RNA from the different experimental conditions. Table 2 indicates CT numbers and the percentage and fold change from the initial inoculum (1.0 x 10^3^).

	N-gene CT # for Biofilm + MHV on SS	N-gene CT # for Biofilm + MHV on PVC	N-gene CT # for Biofilm + MHV on Tile
Control	0; 0	0; 0	0; 0
Biofilm+MHV	14.27 (+3.9%, 1.04-fold); 16.47 (+11.7%, 1.12-fold).	14.47 (+12.0%, 1.12-fold); 16.82 (+12.0%, 1.12-fold).	24.23 (+2.8%, 1.03-fold); 19.87 (+1.5%, 1.02-fold).
Biofilm-MHV	0; 0	0; 0	0; 0
MHV-Biofilm	13.73; 14.74	12.92; 15.02	23.57; 19.57

### MHV survival was significantly inhibited in the presence of biofilms on all surface materials

**Fig 3:**
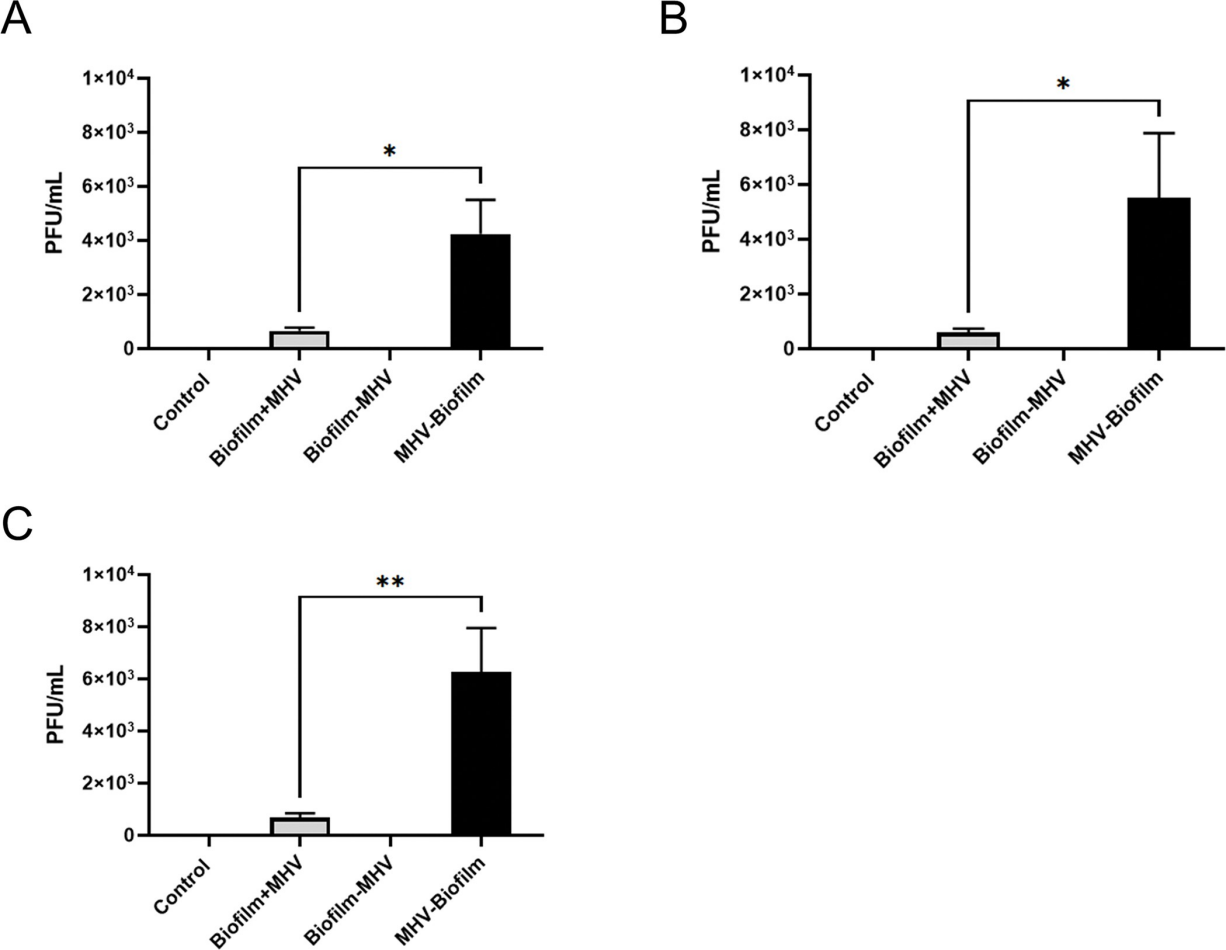
Plaque assay results from biofilm + virus and virus samples on stainless steel, PVC, and ceramic tile chips. (A-C) Results from plaque assays on samples collected from (A) stainless steel, (B) PVC, and (C) tile chips. Each sample was filtered through a 0.2 μm filter and pipetted onto L2 cells in duplicate. Results in this figure are the mean values and standard deviations (error bars) from two independent experiments. Statistical significance was analyzed by unpaired t-test. *: *p* < 0.05; **: *p* < 0.01.

Although RT-qPCR analysis measures viral RNA presence in a quantitative fashion, it does not provide any information on the viral viability; therefore, to identify whether MHV was able to survive and remain infectious when incubated with an environmental biofilm, we performed plaque assays. For all of the materials tested, a significantly higher average of PFU/mL was detected in the absence of biofilm (Table 3), specifically Biofilm+MHV on SS chips gave 650 PFU/mL, compared with 4250 PFU/mL without (Fig 3A, Table 3), representing a 6.54-fold decrease in the presence of the biofilm. On the PVC test surface, the mean PFU/mL for Biofilm+MHV was 600 PFU/mL, compared with 5500 PFU/mL for MHV-Biofilm (Fig 3B, Table 3), equating to a 9.17-fold decrease. Similarly, Biofilm+MHV on ceramic tile gave 675 PFU/mL, whereas the average for MHV-Biofilm on ceramic tile was 6250 PFU/mL (Fig 3C, Table 3), a 9.26-fold difference and very similar to what was observed for PVC. These observations suggest that infectivity/viability of MHV is drastically reduced in the presence of the environmental biofilm and reflect what was identified by RT-qPCR.

**Table 3:** Data from the plaque assay analysis on the recovered MHV viral particles incubated with biofilm. Table 3 indicates PFU/mL numbers and the percentage and fold change from the initial inoculum (1.0 x 10^3^ CFUs).

	PFU/mL for Biofilm + MHV on SS	PFU/mL for Biofilm + MHV on PVC	PFU/mL for Biofilm + MHV on Tile
Control	0; 0	0; 0	0; 0
Biofilm+MHV	7.50 x 10^2 (-566.7%, 6.67-fold); 5.50 x 10^2 (-536.4%, 6.36-fold).	5.00 x 10^2 (-1200%, 13-fold); 7.00 x 10^2 (-542.9%, 6.43-fold).	8.00 x 10^2 (-525.0%, 6.25-fold); 5.50 x 10^2 (-1263.6%, 13.64-fold).
Biofilm-MHV	0; 0	0; 0	0; 0
MHV-Biofilm	5.00 x 10^3; 3.50 x 10^3	6.50 x 10^3; 4.50 x 10^3	5.00 x 10^3; 7.50 x 10^3

## Discussion

The National Cattlemen’s Beef Association has predicted that the cattle industry will potentially experience a $13.6 billion loss due to the novel coronavirus SARS-CoV-2, impacting ranchers across the USA [30]. Much of this loss stems from the rampant spreading of SARS-CoV-2 among plant workers, resulting in the closure of many meat processing plants [31–33]. This created a bottleneck in the supply chain between the livestock producers, feedlot operators and processors resulting in a breakdown in the USA’s meat supply during 2020 and 2021 [34–36]. Additionally, the conditions within meat processing plants could be a conducive environment for SARS-CoV-2 to remain stable for a prolonged period of time (longer than five days). SARS-CoV-2 has been shown previously to remain stable for several days on stainless steel, copper, plastic, PVC and cardboard [23], which are commonly used materials throughout the farm-to-plate chain [24]. Our data confirmed that MHV shows a similar recalcitrance when tested on SS, PVC, and ceramic tiles. Meat processing plants are maintained at 4–7°C and SARS-CoV-2 virions are known to remain stable at colder temperatures [37, 38]; we demonstrated that MHV, which as a mammalian pathogen could be assumed to be strongly adapted to the host body temperature, was very stable on the test surfaces at 7°C for five days. Hence these data suggest that meat processing facilities are at a high risk of harboring and transmitting SARS-CoV-2.

In addition to meat processing facilities being a favorable environment for SARS-CoV-2 to persist and spread, these plants are also highly susceptible to biofilm growth [39–41]. Biofilms form in many different zones throughout food processing plants, including floors, drains, difficult to clean surfaces such as the back of the conveyor belts, and pipes [42, 43]. Often surfaces become hot spots for biofilm development due to poor accessibility for regular hygiene and sanitation maintenance [44]. Furthermore, nearly all biofilms in food processing environments consist of multiple species of microorganisms [43]. The complex interactions within the biofilm community significantly influence the architecture, activity, and sanitizer tolerance of the biofilm [41, 43, 45, 46]. Biofilms could potentially help SARS-CoV-2 spread throughout the meat processing facility and offer protection to the virus particles by either phagocytosing or endocytosing the virus particles that attach to the surface of the bacteria and protect them from being degraded by surface sanitizers used in the meat processing facility [20, 41, 47, 48]. Once the virus particles are inside of the bacteria, the bacteria could spread the virus throughout the meat processing facility through the water drainage system or from bacteria that are spread throughout the meat processing facility via the heating, ventilation, and air conditioning (HVAC) system [49–54].

In our study, we utilized MHV as a surrogate for SARS-CoV-2, as it has been shown to be an appropriate model for assessing methods and performance of SARS-CoV-2 but in a Biosafety Level 2 workspace [55]. Floor drain samples from a meat processing plant were used to epitomize the microbial community found in these settings. As floor drains collect all rinsing water and liquid wastes in the plant, the microbial communities from the floor drains have been considered to represent all microecological niches that encompass the various microorganisms within the processing plant environment [41, 45]. We investigated the survival rates of MHV on commonly used materials in the meat processing facilities: SS, PVC, and ceramic tiles. We observed that MHV was able to remain present and viable on all the materials tested but persistence was reduced in the presence of biofilm, as indicated by the plaque assays (Figs 2 & 3, Tables 2 & 3).

Despite there being several environmental parameters that could have facilitated the survival of MHV in the viral-environmental biofilm [23], plaque forming units (PFU/mL) and viral RNA, as measured by RT-qPCR, were both significantly reduced when MHV was exposed to an environmental biofilm compared to when MHV was inoculated on the materials by itself (Fig 2, Table 2). When compared with the plaque assay data, our data indicates that the majority of the Biofilm+MHV signal from the RT-qPCR data was from non-viable/inactive virus. The PFU/mL recovered from virus incubated directly from the different materials, in the absence of biofilm, indicated a reduction of 37.5–57.5% of the initial infectious viral particles, however, from the biofilms, we observed approximately a 94% loss in infectivity. As virus is unable to replicate outside of a host cell we did not expect to observe numbers higher than the initial inoculum (1x10^3^ PFUs) for our plaque assays, however, the reduction in numbers was stark in the presence of biofilm, in comparison to the numbers returned in the absence [56]. Nonetheless although we saw a large reduction in viral numbers in the presence of biofilm, we did observe MHV survival in all the test conditions, and given the highly infectious nature of SARS-CoV-2, particularly for some of the variant strains such as omicron, even a residual level remaining viable on surfaces represents a potential health hazard [56–58].

One of the most interesting observations from this study was an increase in biofilm biovolume in the presence of MHV ranging from a 1.74–2.11-fold increase (Fig 1, Table 1). This result is in concordance with the findings from other work on biofilms, where virus particles also enhanced the biovolume of the biofilm [25, 59–61]. In nature, microbial communities survive as mixed-species communities with complex interactions with eukaryotes and prokaryotes living in a complex biofilm web [20, 22, 62]. The increase in biovolume could be due to the triggering of a defense mechanism due to the biofilm identifying the presence of a foreign substance and reacting by increasing its biovolume to try and spread out and obtain as many resources as possible (Fig 1). Therefore, this finding is critical in deciphering the role of viral interactions with biofilms in harboring the virus particles. We tested viral viability on surfaces with and without biofilms but without the inclusion of any disinfectant; in a real-life situation, it could well be that virus persistence on surfaces would be lower than in biofilms when disinfectants were included. Previous studies have indicated that biofilms may provide a physical barrier protecting the antimicrobial agent from coming into contact with the virus particles [22, 26]. Given the impact of SARS-CoV-2 on meat processing facilities, it is vital to understand the role that the biofilm is serving in protecting and harboring the virus.

From our viability studies with biofilm and virus, we observed that MHV was able to remain infectious when mixed with an environmental biofilm on SS, PVC, or on ceramic tile (Fig 3, Table 3), but was considerably more infectious when incubated on these same surfaces without biofilm [18]. Higher numbers of MHV were recovered from PVC and ceramic tile than on SS. These results could be due to the slick surfaces of the PVC and ceramic tile which potentially could allow for better removal of the virus following incubation, in comparison to the SS which has a rough surface [18, 51, 63]. The infectivity of MHV when mixed with the environmental biofilm was approximately the same between SS, PVC, and ceramic tile, but we observed a significant loss in infectivity for MHV overall following exposure to biofilm. This could be due to some of the virus being incorporated as a part of the biofilm, and therefore, would have been removed from the filtrate when each sample was filtered prior to the plaque assay. Another potential reason is that the biofilm contains exogenous proteases and nucleases which result in a degradation of the viral particles, leaving residual RNA fragments which are measurable by qPCR, which fits with the data we observed. Further work using long-read based NGS technology to sequence the entire biofilm would provide further information on the integrity of the viral RNA within the biofilm.

These data suggest that SARS-CoV-2 could easily remain viable on the materials tested, which are commonly found in meat processing (SS, PVC, and ceramic tile) for many days, Microbial biofilms could be considered as potential reservoirs of pathogenic viruses, indeed, they are probably responsible for numerous persistent viral infections [22]. Although we observed increased viral viability in the absence of biofilm, there is the potential that viral particles could travel through that liquids that seep through the drains in these facilities and survive along with the environmental biofilms found in the drains, where the biofilm matrix would provide a physical barrier protecting the SARS-CoV-2 particles from sanitizers, which has been previously observed for other viruses [64, 65]. Microbial communities in the biofilm could also enhance the dispersal of viruses through several active processes: viruses can bind to flagellated bacteria and be transported over relatively large distances by bacterial motility [26, 66]. Even without binding to the virus, bacteria can generate strong physical forces that result in mixing of surrounding fluids [21, 62], thus bacteria can colonize new areas by continuously producing new extracellular matrix (ECM) and actively swarming outwards, creating a drag and moving the virus too [41, 45]. These two bacterial mechanisms could create new locations throughout the meat-processing facility which can harbor SARS-CoV-2.

SARS-CoV-2 is well documented to spread through aerosols [13, 23]. One common process in meat processing facilities involves washing the floors with pressurized water, this results in a huge release of aerosolized particles, and if SARS-CoV-2 is present this will become aerosolized too. Once the virus is airborne, the colder temperature and the airflow from HVAC systems in the meat processing facility will facilitate the survival and spread of SARS-CoV-2 throughout the plant, in agreement with recent fluid mechanics simulations [67].

The above findings lead us to conclude that both the recalcitrance of coronaviruses on surfaces, the documented survival at low temperatures, the presence of multi-species biofilms, and HVAC systems moving aerosols could all come together to play a significant indication for the higher rates of COVID-19 cases in meat processing facilities. Due to the nature of the work, it is difficult to implement workplace physical distancing. This, on top of other factors recognized among meat processing frontline workers such as crowded living and transportation conditions [68] have all converged to create a quagmire where SARS-CoV-2 is rapidly transmitted. Continued studies on SARS-CoV-2 in meat processing plants will help provide new information on how we understand the relationship between viruses, bacteria, and fungi. This knowledge will help design new engineering and workflow strategies for inhibiting future outbreaks of SARS-CoV-2 and other pathogens within these facilities.

## Conclusions

Our data provides evidence that MHV (which was adopted as a surrogate for SARS-CoV-2) can persist for up to five days post-inoculation under the environmental conditions (temperatures and surfaces) typically found in meat processing plants. Although we observed a lower survival rate from MHV in biofilms, compared to on surfaces alone, our results suggest that environmental multi-species biofilms from meat processing plants can harbor viral particles, potentially protecting the virus from sanitizers and facilitating a reservoir for SARS-CoV-2 to persist and periodically disperse throughout the meat processing facility. We also observed that biofilms were stimulated by MHV, and this resulted in an increase in biovolume. Further studies will be required to decipher the molecular mechanisms of viral:biofilm interactions, both from the perspective of viral protection and survival, but also from the bacterial sensing and reacting to the virus side. Together, this knowledge will help in designing targeted prevention strategies to eradicate the harborage and spread of viral lodged multi-species biofilms.

## Materials and methods

### Drain sample collection and characterization

Meat processing floor drain biofilms were collected following the previously described protocol [41] and were generously provided for this study by Drs. Mick Bosilivac and Rong Wang USDA-ARS-USMARC, Clay Center, Nebraska. The biofilm used in this study was collected from a floor drain from the cooler of a single meat processing facility. The biofilm used in this study consisted of the following dominant taxa of Flavobacteriacea, Moraxellacea, Listeriacea, Pseudomonacea, Enterobacteriaceae, Weeksellacea, Sphingobacteriaceae, and Aeromonadaceae [41].

### Cell lines and MHV propagation

MHV strain A59 (ATCC® VR-764) was used for all of the experiments in this study as a BSL-2 surrogate model to study SARS-CoV-2 persistence in meat processing plants. L2 cells (ATCC® CCL–149TM) were used for MHV plaque assay. In addition, the mouse asterocytoma-derived cell line (DBT), was used to propagate MHV (generously provided by Dr. Julian Leibowitz, Texas A&M Health Science Center, College Station, TX). All cells used in this study were cultured at 37°C in 5% CO_2_ in T-175-cm^2^ ventilated-cap flasks with 25 mL of Dulbecco’s modified Eagle medium (DMEM; Cellgro) supplemented with 10% fetal bovine serum (FBS), penicillin (50 IU/mL), and streptomycin (50 μg/mL).

The virus stocks used for this study were produced as previously described [69], with the addition of 1% Penicillin/Streptomycin. Specifically, MHV stocks were produced by seeding 1.5 x 10^7^ DBT cells in four T-175-cm^2^ ventilated-cap flasks with 25 mL of D10 media (DMEM+10% FBS and 1% Penicillin/Streptomycin mixture) in each flask and incubated for 24 hours at 37°C with 5% CO_2_. After the 24 hour-incubation period, each flask was inoculated with MHV to produce a multiplicity of infection (MOI) between 0.1 and 0.001 PFU/cell, and incubated for 24 hours at 37°C with 5% CO_2_. Following incubation, the flask was frozen at -80°C for at least one hour to help dislodge the cells from the flask. The flasks were then thawed and the cell suspension transferred into a 15-mL polypropylene screw-cap tube, and sonicated on ice in a cup sonicator at 100 W with three bursts of 20 seconds, resting on ice for 20 seconds between each burst. The cells were collected by centrifuging the tube for 10 minutes at 3000 RPM at 4°C, and the virus stock aliquoted into 0.5-mL portions in 1 mL screw-cap freezer tubes and stored at -80°C. One aliquot of the virus was used determine the titer of the virus via plaque assay.

### Assay of MHV infectivity

The viral infectivity for each virus was determined by titrating each virus stock onto cultured L2 cells and a solid double overlay plaque assay was performed as previously described [70]. The MHV viral titer used for all experiments was 1.0 x 10^4^ PFU/mL, and 100 μL (1x10^3^ viral particles) were used for each sample tested.

### Biofilm formation with drain sample bacteria and MHV

**Fig 4:**
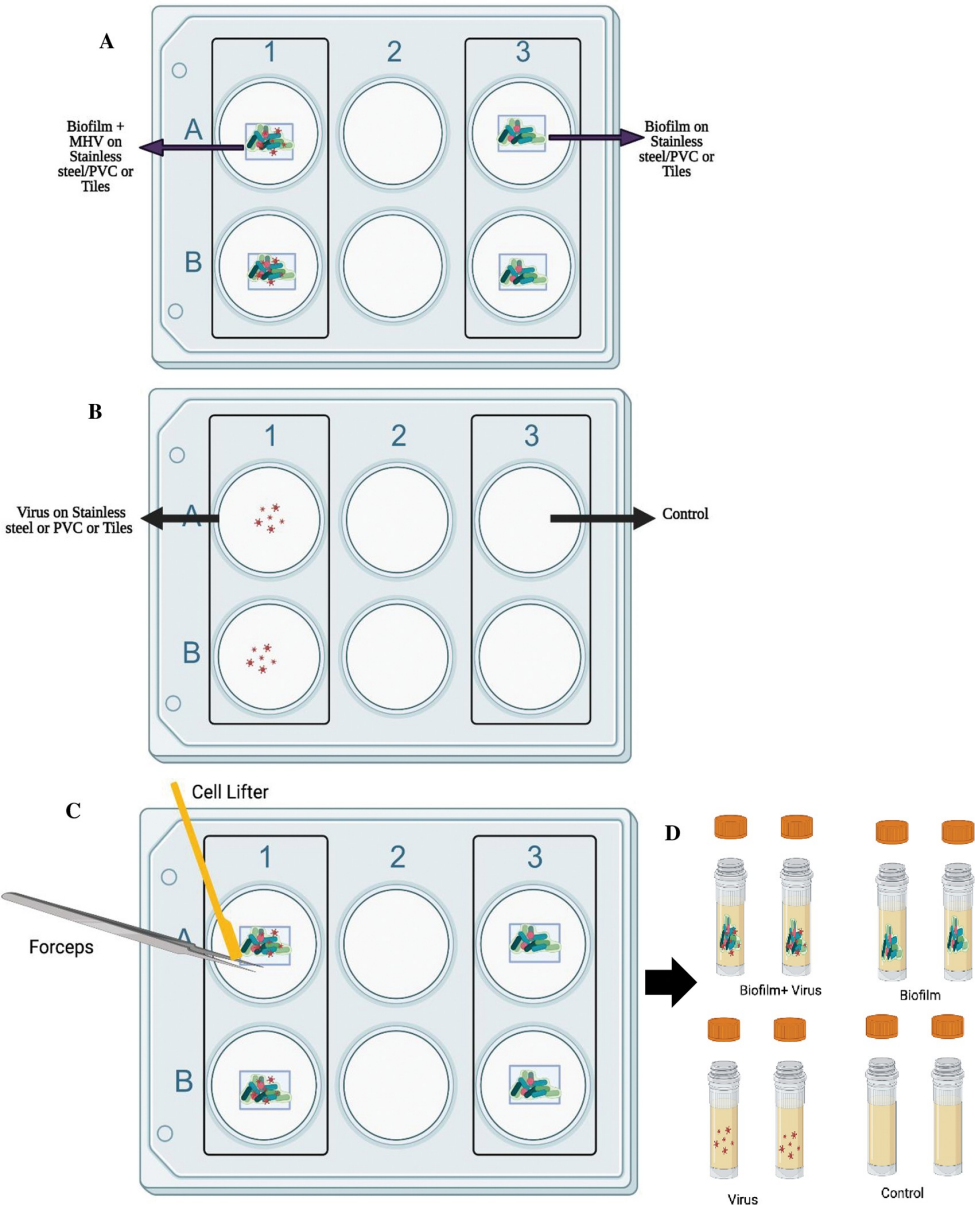
Schematic representation of floor drain biofilm and virus experiment. (A and B): Experimental set up with Biofilm + Virus, Biofilm, Virus, and Negative Control in duplicate. The experimental set is incubated at 7°C for 5 days. (C). After 5 days, the biofilm was harvested from SS, PVC, or tile chips using a cell lifter and forceps and rinsed with 1 mL of LB-NS. (D) Harvested cells were stored in a screw-cap tube at -80°C until assayed.

Floor drain samples were pre-cultured by inoculating 1:50 into Lennox Broth without salt medium (LB-NS, Acumedia Manufacturers, Baltimore, MD) and incubated at 7°C for five days with orbital shaking at 200 rpm [41]. On the fifth day, a 1.0 mL aliquot was removed from each sample, diluted in sterile LB-NS medium, and plated onto Trypticase soy agar (TSA) plates for colony enumeration after overnight incubation at 37°C. To investigate whether biofilms formed from floor drain samples can support the harborage of MHV, biofilms were developed with or without MHV on SS, PVC, and ceramic tile (Fig 4). Controls were included on all the test surfaces. These were MHV alone (no biofilm) and a media only negative control. The experiments were set-up in duplicate in 6-well tissue culture plates. Each well contained one sterile (18X18X2 mm) SS, PVC, or ceramic tile chip. The following test combinations were added to the top facing surface of the chip: (A) Biofilm+MHV: contained 100 μL of the five-day floor drain pre-culture (described above) mixed with 100 μL of MHV in DMEM and 100 μL of LB-NS media;(B) Biofilm-MHV contained 100 μL of the five-day biofilm pre-culture, 100 μL of DMEM, and 100 μL of LB-NS media; (C) MHV alone comprised of: 100 μL of MHV (1x10^3^) in DMEM and 200 μL of LB-NS media; (D) Media only control (Negative Control) was: 100 μL of DMEM and 200 μL of LB-NS. Each experimental variable was incubated at 7°C for five days. At the end of the incubation period, biofilm biomass/virus was harvested from each chip by lifting the chip with sterile forceps, scraping the material on both sides with a sterile cell scraper into a sterile tube and rinsing the chip with 1 mL of LB-NS, which was also collected (Fig 4). The collected sample was homogenized by pipetting. The drain biofilm biomass was determined by taking 100 μL of the homogenate, performing 10-fold dilutions into LB-NS and plating on TSA plates for colony enumeration following an overnight incubation at 37°C. The remaining homogenate was used for qPCR and plaque assay analysis. Results from this experiment are the mean values and standard deviations (error bars) from two independent experiments.

### MHV RT-qPCR analysis

Viral RNA from each sample was extracted and purified to perform RT-qPCR to determine the relative copy numbers of MHV in each sample. Viral RNA was extracted using NEB’s Monarch Total RNA Miniprep Kit and following the Tough-to-Lyse manufacturer’s protocol. Purified RNA samples were quantified by using a Thermo Fisher Scientific ND-1000 spectrophotometer. Purified RNA samples were stored at -20°C. Taqman-based RT-qPCR analysis was carried out using NEB’s Luna^®^ Universal Probe One-Step RT-qPCR kit. Purified RNA extracted from MHV was used for the positive control and to create a standard curve. The RT-qPCR reactions were completed in 25 μL volumes using the Luna Universal Probe One-Step Reaction Mix. The RT-qPCR mixture contained 10 μL of Luna Universal Probe One-Step Reaction Mix, 1 μL of Luna WarmStart RT Enzyme Mix, 400 nM of forward primer (5’-GGAACTTCTCGTTGGGCATTATACT-3’), 400 nM of reverse primer (5’- ACCACAAGATTATCATTTTCACAACATA-3’), 200 nM of probe (IDT) (5’-FAM- ACATGCTAC-ZEN-GGCTCGTGTAACCGAACTGT-3IABkFQ-3’), 250 ng RNA, and nuclease free water [71]. The RT-qPCR analysis was performed using a Bio-Rad CFX96 Deep Well Real Time thermal cycler. Reverse transcription occurred at 55°C for 10 minutes, after which there was a denaturation and *Taq* polymerase activation step at 95°C for 1 minute, and then 40 cycles at 95°C for 15 seconds followed by 60°C for 30 seconds for data collection. RT-qPCR reactions were performed in quadruplicate for each sample and the sample threshold cycle (CT) was used for data analysis. Gene copy numbers were calculated by comparing the CT value for 250 ng of MHV on the standard curve with the CT value for each sample. The following equation was used to calculate the gene copy numbers: Gene Copy Number = (Copy Number of 250 ng of positive control)–((CT Pos Cont.–CT exp cont)/CT exp cont)*(Copy number of 250 ng of positive control) [72]. Data for each sample was compared using positive and negative controls performed in duplicate. Results from this experiment are the mean value and standard deviation (error bars) from two independent experiments.

### Plaque assay analysis

Plaque assays were performed to determine MHV infectivity/viability. 300 μL of each recovered sample homogenate was filtered through a 0.2 μm syringe filter, to remove bacterial contaminants, before being serially diluted in DMEM with 2% FBS and 1% Streptomycin/Penicillin mix. Each sample was plated in duplicate. Results from this experiment were obtained from two independent experiments (biological replicates). A previously published protocol was followed for the double layer overlay plaque assay [72].

## Supporting information

S1 Data (XLSX)

S2 Data (XLSX)
